# Dieulafoy’s disease of the bronchus: a possible mistake

**DOI:** 10.1186/2049-6958-7-40

**Published:** 2012-11-08

**Authors:** Emanuela E Barisione, Gabriele G Ferretti, Silvia S Ravera, Mario M Salio

**Affiliations:** 1 SS. Antonio e Biagio e C. Arrigo Hospital, Alessandria, Italy

**Keywords:** Dieulafoy’s disease, Massive hemoptysis, Vascular lesion

## Abstract

We present a case of a 57 year old woman who suffered from massive hemoptysis; she was sent to our Department for a suspect neoformant lesion. We assumed it might be a Dielafoy’s disease and proceeded with an imaging study that confirmed the diagnosis. After embolization the patient no longer showed signs of bleeding. In brief, we concluded that whenever there is a suspect of Dielafoy’s disease, the biopsy has to be avoided.

## Background

Dieulafoy’s disease of the bronchus is supposed to be a very rare disease. In fact only few cases are reported in literature [4;7]. This condition should be clinically suspected in heavy smokers with recurring and unexplained episodes of massive hemoptysis. The bleeding can occur immediately after the biopsy or delayed up to 12 days. The diagnosis can be made through imaging. Angiographic images document that this vascular malformation is based on a left-to-right shunt, with a bronchial artery draining into a pulmonary artery. Endobronchial ultrasound may be helpful in detecting the vascular nature of the lesion [1].

## Case presentation

**Figure 1:**
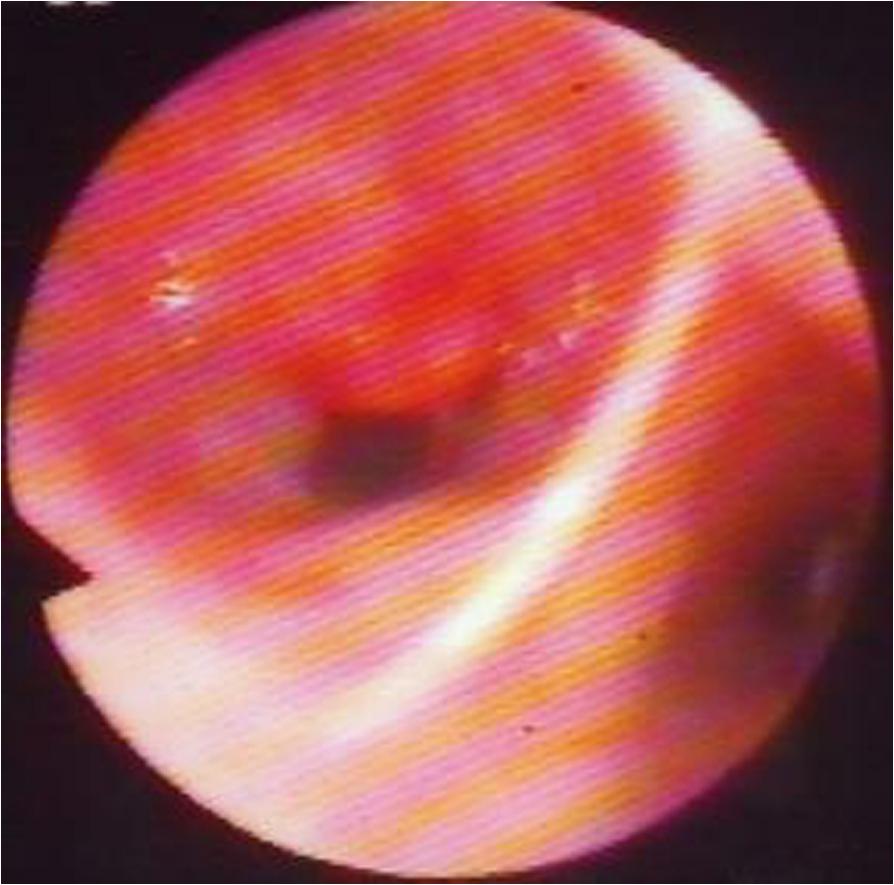
Lesion at the beginning of superior right bronchus.

**Figure 2:**
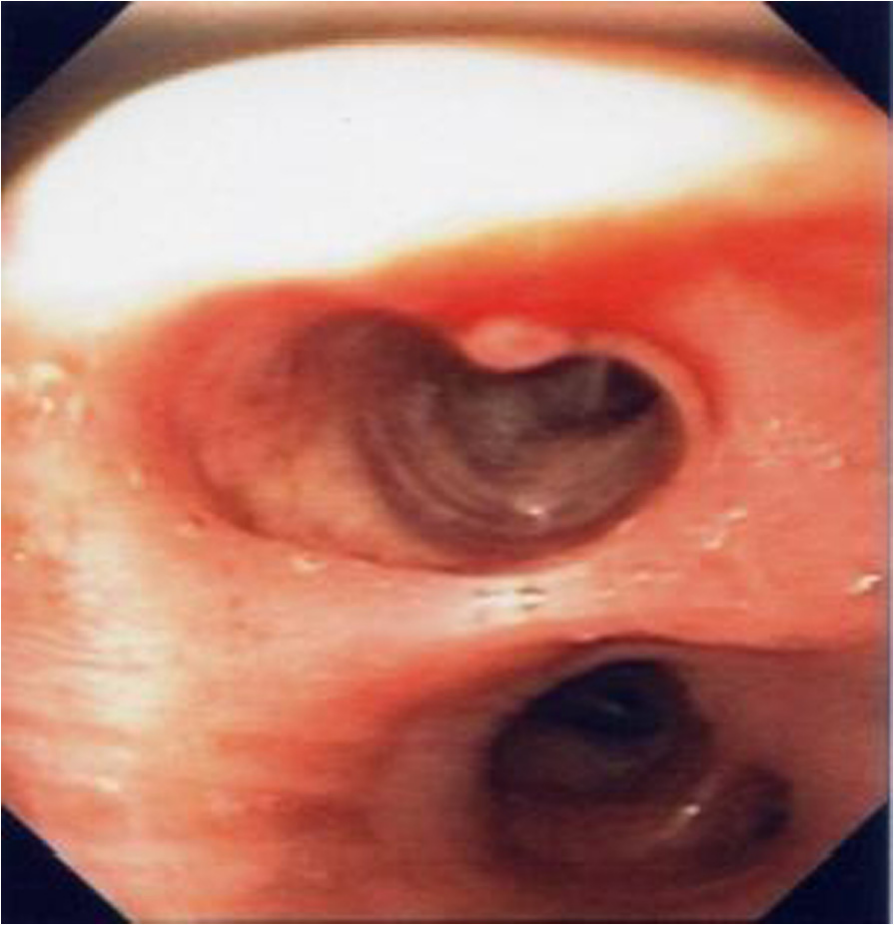
Lesion at the beginning of medium bronchus.

**Figure 3:**
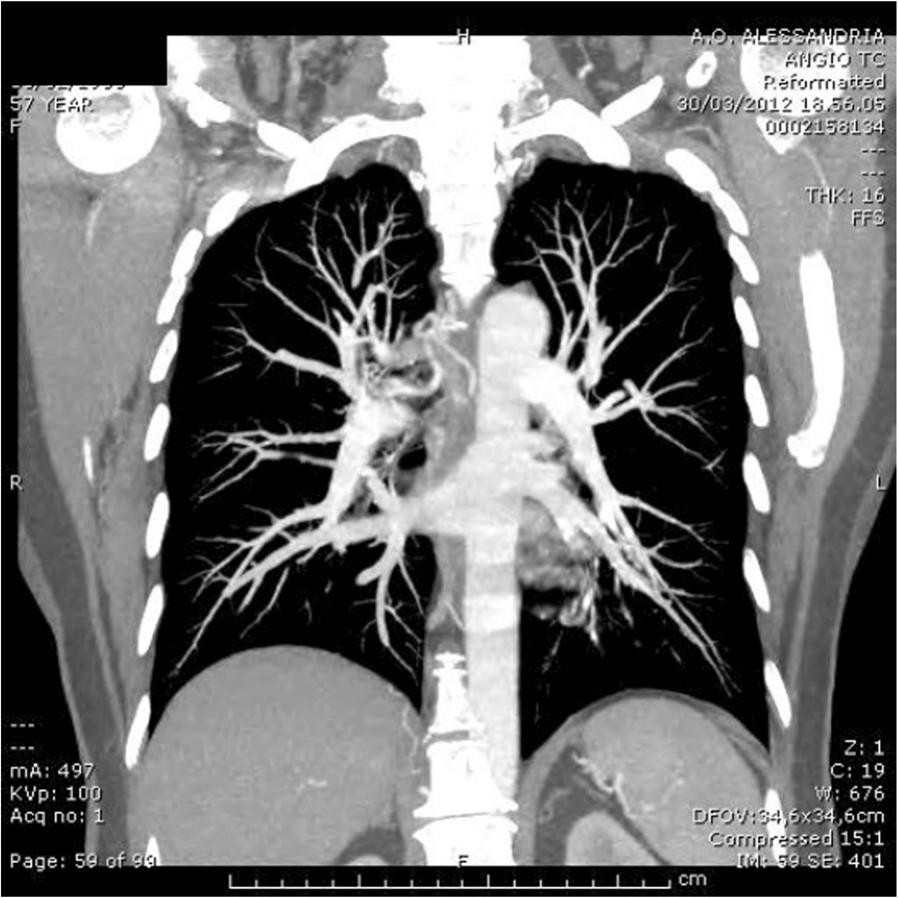
Arteriography.

**Figure 4:**
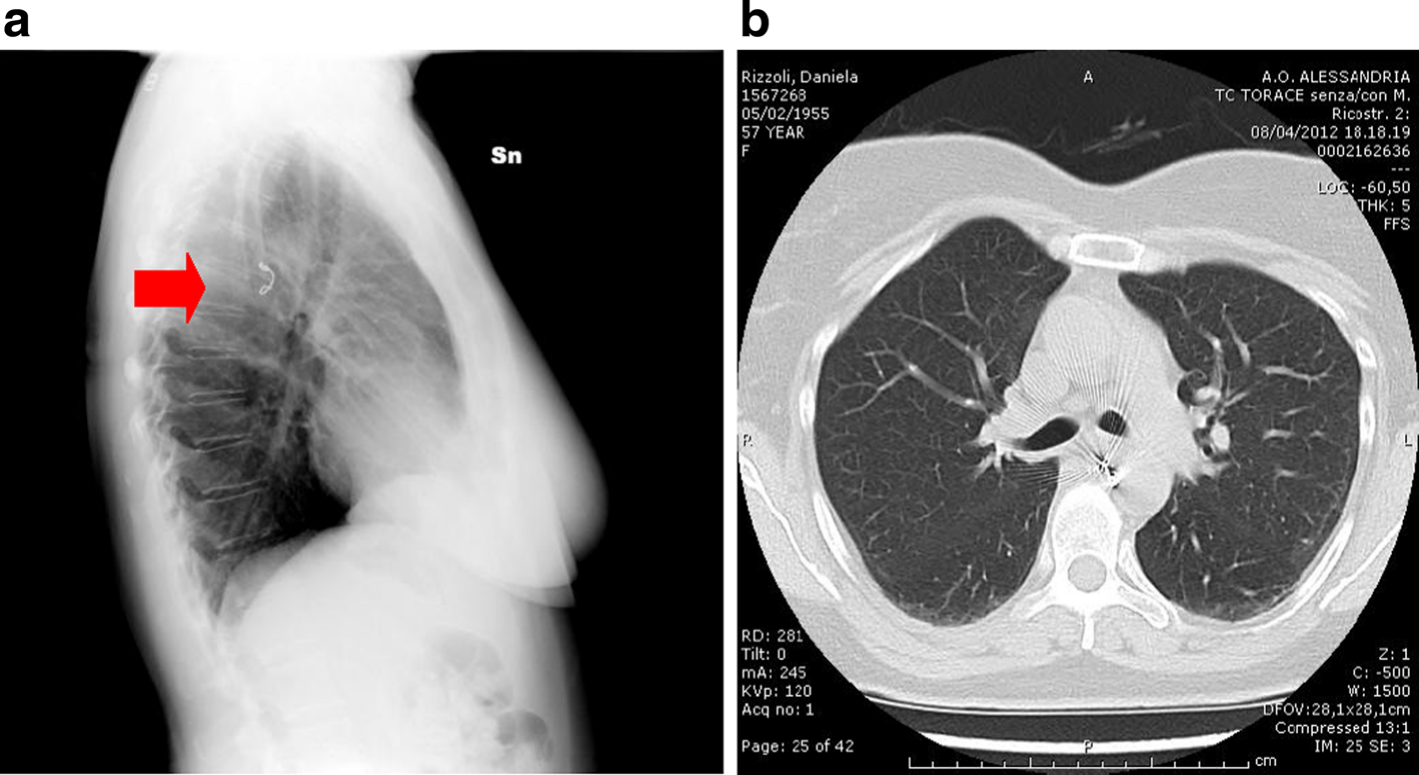
(Black arrow): Spiral in the right bronchial artery, [a: X-ray b: CT].

A 57 year old woman, non-smoker, non-atopic, was sent to our Department and we detected a neoformant lesion at the beginning of the superior right bronchus (Figure 1). Previously she had been admitted to another hospital (in February 2012) after seven episodes of massive hemoptysis. At the bronchoscopy there was no blood in the bronchial tree, but a little lesion with normal mucosa in the superior bronchus. The biopsy was followed by a massive hemoptysis that stopped only after 4 doses of tranexamic acid 5ml/500mg. During the emergency the patient presented a hypotensive crisis, such as after the bleeding cessation she was transferred to the intensive care unit for hemodynamic monitoring. One hour later another bronchoscopic examination was performed confirming the bleeding had stopped. In March a CT/PET was practised and proved negative for hypercaptations. The histological evidence of the biopsy showed normal bronchial mucosa with conserved structure, so this report was considered negative for neoplastic lesions. The patient arrived at our hospital at the end of March, because doctors in charge had suggested a biopsy should be carried out with a rigid bronchoscope which is safer in case of bleeding. However, after taking view of the histological description and visual image of the previous bronchoscopy we decided to use a flexible bronchoscopy in the presence of an anaesthesiologist. We found a lesion of about 1–2 mm in diameter at the beginning of the medium bronchus (Figure 2); it raised from the surface with a white cap and covered form, with apparently normal mucosa. There was no lesion in the right upper bronchus, probably because it had disappeared after the previous biopsy. Suspecting a Dieulafoy’s disease, we didn’t carry out a further biopsy of the lesion and proceeded to an angiographic study. The arteriography showed convoluted and ectatic bronchial vascular structures, particularly around and behind the trachea and around the right bronchus (Figure 3). An embolization of the right bronchial artery and in particular of the common tract of the intercostal bronchial trunk was then performed using three 5mm spirals (Figure 4).

## Discussion

Dieulafoy’s disease is an extremely rare vascular anomaly, characterized by the presence of a dysplastic artery in the sub-mucosa. At present, there are few proven cases reported in literature [2-4]. The pathogenesis of Dieulafoy’s disease remains unclear. This anomaly was first reported in the gastrointestinal tract [5]; more recently it has been described also in the respiratory tract [6]. While in the gastrointestinal tract the bleeding is often spontaneous but also fatal, in the bronchial tree profused bleeding often occurs after a biopsy. However, cases of spontaneous bleeding have also been described [3]. It is still unknown whether the origin of the anomaly is congenital or acquired, but age and tobacco use are thought to have an influence on the occurrence of the disease [2]. The trigger factor of the vessel rupture is unknown. Furthermore, the nature of the bleeding vessel remains controversial. Dieulafoy’s disease of the bronchus is probably underestimated. Massive hemoptysis is a life threatening condition associated with a mortality rate exceeding 50% in the absence of adequate treatments [7,8]. The characteristics of the lesion are non-specific, but in the presence of a small (usually <1 cm), sessile, non pulsating nodular lesion, often with a white cap, and apparently normal mucosa, Dieulafoy’s disease should be taken into account. The respiratory epithelium shows focal squamous metaplasia and diffused thickening of the basal membrane. In bronchial Dieulafoy’s disease, selective embolization has been suggested as a method for stopping the bleeding [9,10] and only in few cases the patient requires surgical resection [11].

## Conclusions

In brief, Dieulafoy’s disease of the bronchus is more frequent than thought, so this diagnostic option should be considered when there is a patient with recurring massive hemoptysis, which cannot be explained otherwise. Obviously, in this case the biopsy has to be avoided even when no active bleeding is evident.

## Consent

Written informed consent was obtained from the patient for publication of this case report and any accompanying images.

## Competing interests

The authors declare that they have no competing interests.

## Authors’ contributions

EB wrote the case report and revised the literature, GF participated to choose the images, SR participated to revise the literature, MS chose the images and performed the flexible bronchoscopy; All authors read and approved the final manuscript.
